# Bayesian Optimization-Assisted Screening to Identify Improved Reaction Conditions for Spiro-Dithiolane Synthesis

**DOI:** 10.3390/molecules28135180

**Published:** 2023-07-03

**Authors:** Masaru Kondo, Hettiarachchige Dona Piyumi Wathsala, Kazunori Ishikawa, Daisuke Yamashita, Takeshi Miyazaki, Yoji Ohno, Hiroaki Sasai, Takashi Washio, Shinobu Takizawa

**Affiliations:** 1 SANKEN, Osaka University, Ibaraki-shi 567-0047, Japan; masaru.kondo.fg74@vc.ibaraki.ac.jp (M.K.); washio@ar.sanken.osaka-u.ac.jp (T.W.); 2 Department of Materials Science and Engineering, Graduate School of Science and Engineering, Ibaraki University, Nakanarusawa-cho, Hitachi-shi 316-8511, Japan; 3 Asahi Chemical Co., Ltd., Mitsuya-Minami, Yodogawa Ward, Osaka-shi 532-0035, Japany_ohno@chem-asahi.co.jp (Y.O.); 4 Graduate School of Pharmaceutical Sciences, Osaka University, Suita-shi 565-0871, Japan

**Keywords:** Bayesian optimization (BO), Gaussian process regression (GPR), random screening, Latin hypercube sampling (LHS), spiro-dithiolane, process chemistry, scalable synthesis

## Abstract

Bayesian optimization (BO)-assisted screening was applied to identify improved reaction conditions toward a hundred-gram scale-up synthesis of 2,3,7,8-tetrathiaspiro[4.4]nonane (**1**), a key synthetic intermediate of 2,2-bis(mercaptomethyl)propane-1,3-dithiol [tetramercaptan pentaerythritol]. Starting from the initial training set (ITS) consisting of six trials sampled by random screening for BO, suitable parameters were predicted (78% conversion yield of spiro-dithiolane **1**) within seven experiments. Moreover, BO-assisted screening with the ITS selected by Latin hypercube sampling (LHS) further improved the yield of **1** to 89% within the eight trials. The established conditions were confirmed to be satisfactory for a hundred grams scale-up synthesis of **1**.

## 1. Introduction

Screening and optimization of reaction conditions are essential processes in the field of organic synthesis [1]. Although these processes are important for saving resources, such energy, chemicals, time, and manpower, to synthesize products with higher yield and selectivity, the conventional trial-and-error methodology is limited to the optimization of many parameters in a small number of experiments for the manufacturing chemical processes, even if in bench-scale synthesis. Thus, efficient screening and optimization of a small number of trials are highly demanded in the field of process chemistry. To accelerate reaction optimization in industry, a design of experiment (DoE) [2,3] and real-time optimization [4,5] have often been employed. Over the past few decades, machine-learning (ML) has emerged as a robust and reliable tool that can be used to achieve efficient optimization. Recently, ML and data science have been extensively developed for chemical manufacturing [6,7,8].

Bayesian optimization (BO) is an effective data-driven probabilistic method for predicting the maximum of a black-box objective function using the Bayesian approach with a dataset collected in the previous sampling and ML modeling iteration [9,10]. BO estimates the next parameters to be examined on the basis of modeling of a collected experimental dataset by Gaussian process regression which is a kernel-based non-parametric regression method and maximizing the acquisition function. Subsequently, the predicted parameters are assessed by conducting a practical experiment. Afterwards, a new data point is adopted for the aforementioned BO process. Finally, the most suitable reaction conditions leading to a suitable outcome are predicted through repetition of the BO estimation and experimental evaluation. BO not only seeks a probable maximum (*exploitation*) but also avoids a local maximum by exploring locations where the prediction certainty is insufficient (*exploration*).

In recent, BO has attracted increasing attention in the industrial chemistry field. Kaneko et al. simulated the entire ethylene oxide production process using BO [11]. Lee, Zahid, and co-workers also found that BO is useful for designing the reactor for the large-scale synthesis of toluene diisocyanate [12]. Chachuat et al. investigated real-time optimization using BO to overcome the plant-model mismatch [13]. In the other fields, BO has been used to rapidly reach suitable conditions for the lab-scale synthesis of fine-chemicals [14,15,16,17,18,19,20]. Our group also demonstrated the utility of BO-assisted screening in the electrochemical oxidative synthesis of α-ketiminophosphonates from the corresponding α-amino phosphonates [16], multiparameter screening of the electrochemical reductive carboxylation of imines to α-amino acids in a flow microreactor using BO with consistent [18], and BO-driven parallel screening of multiple parameters of micromixers and organocatalytic conditions in the flow *C*_1_-symmetric biaryl syntheses [19]. These BO exploration results encouraged us to further investigate a large-scale synthesis of industrial products.

Polyvalent mercaptans such as 1,3-dimercaptopropan-2-ol (CAS# 584-04-3), 2,2-bis(mercaptomethyl)propane-1,3-diol (CAS# 19333-66-5), 3-mercapto-2,2-bis(mercaptomethyl)propan-1-ol (CAS# 500283-12-5), and 2-ethyl-2-(mercaptomethyl)propane-1,3-dithiol (CAS# 121318-18-1) are used in both thermo-curing and photo-curing reactions [21]. Owing to this feature, they can be applied to highly refractive resins, coating materials, adhesives, and paints. Mercaptans are useful chain-transfer agents in radical polymerization, as curing agents for epoxy resins, as cleaning agents for electronic parts, and in thin copper-film manufacturing. Among the polyvalent mercaptans, tetramercaptan pentaerythritol, which has a significantly high sulfur content, is used in material science, organic fine chemistry, and even in coordination chemistry as an assembling ligand [22,23,24,25,26]. Grosu et al. synthesized new spiro and trispiro compounds with 2,4,8,10-tetrathiaspiro[5.5]undecane and 7,11,18,21-tetrathia[5.2.2.5.2.2]heneicosane units form tetramercaptan pentaerythritols [23]. Abdel-Razik synthesized organic microporous materials based on silicon-containing spirothioketal polymers via a 1,3-dioxol-forming polymerization reaction between 1,1a,4,4a,5,5a,8,8a-octahydro-2,3,6,7tetra(trimethylsilyl)-9,10-anthraquinone and the tetramercaptan pentaerythritol [24]. Hager et al. applied tetramercaptan pentaerythritol as a crosslinker to develop novel self-healing sulfur-containing polymers [25]. Song et al. prepared the triple butterfly complexes [(*µ*-Ph_2_P)Fe_2_(CO)_6_]_2_[Fe_2_(CO)_6_][(*µ*-SCH_2_)_4_C] and [(*µ-σ,π*-CH_2_CH=CH_2_)Fe_2_(CO)_6_]_2_[Fe_2_(CO)_6_][(*µ*-SCH_2_)_4_C] through a reaction of Ph_2_PCl or CH_2_=CHCH_2_Br with the *µ*-CO containing dianion {[(*µ*-CO)Fe_2_(CO)_6_]_2_[Fe_2_(CO)_6_][(*µ*-SCH_2_)_4_C]}^2–^ formed in situ from tetraanion {[(µ-CO)Fe_2_(CO)_6_]_4_[(*µ*-SCH_2_)_4_C]}^4–^ generated initially by reaction of the tetramercaptan pentaerythritol with Fe_3_(CO)_12_ and Et_3_N [26].

**Figure 1:**
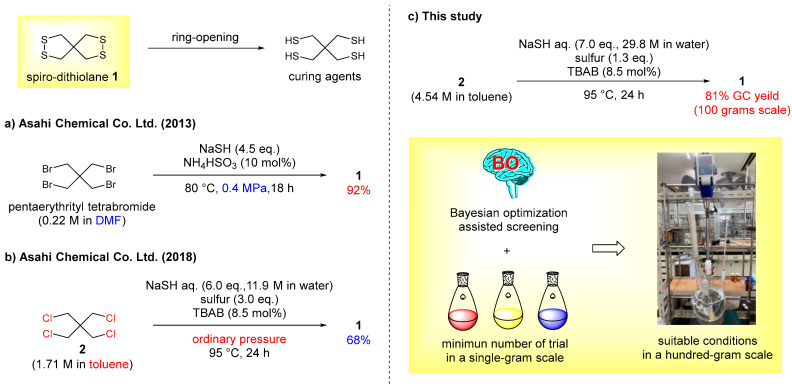
Large-scale synthesis of spiro-dithiolane **1**. (**a**) First-generation synthesis: use of pentaerythrityl tetrabromide in a DMF solution under high pressure [30]. (**b**) Second-generation synthesis: use of pentaerythrityl tetrachloride (**2**) in a toluene solution under ordinary pressure [21]. (**c**) This work: BO established reaction conditions using Latin hypercube sampling (LHS) based on second-generation synthetic conditions.

In general, 2,2-bis(mercaptomethyl)propane-1,3-dithiol (CAS# 4720-60-9) [tetramercaptan pentaerythritol] was prepared via reductive ring-opening reaction of 2,3,7,8-tetrathiaspiro[4.4]nonane (**1**) [27,28]. Spiro-dithiolane **1** was prepared by sulfurization of pentaerythrithiol derivatives with appropriate leaving groups, such as OMs and Br [29,30,31]. In 1992, Chandrasekaran et al. reported the synthesis of spiro-dithiolane **1** from pentaerythrityl tetrabromide (2640 USD/mol from TCI America) using piperidinium tetrathiotungstate as a sulfur transfer reagent in DMF [29]. In 2013, our group independently established the following synthetic procedure of the spiro-dithiolane **1** [30]: pentaerythrityl tetrabromide (0.22 M in DMF), sodium sulfide [4.5 equivalent (eq.)], and catalytic amounts of ammonium hydrogensulfite (10 mol%) were mixed at 80 °C under 0.4 MPa for 18 h, affording **1** in 92% yield (Figure 1a). However, these reactions required stoichiometric amount of the metal reagent [29] and/or lower atom-economical pentaerythrithiol derivatives and expensive DMF as a solvent [29,30]. For suppressing high pressure conditions as well as saving the costs on the industrial process, an alternative synthetic procedure for the plant scale were established by using pentaerythrityl tetrachloride (**2**) (1.71 M in DMF) (1830 USD/mol from TCI America) at ordinary pressure (Figure 1b). However, the obtained yield of the spiro-dithiolane **1** dropped from 92% to 68% (Figure 1a,b).

To re-evaluate and improve the chemical yield of spiro-dithiolane **1**, in this work, BO-assisted rescreening conditions for the synthesis of **1** were conducted (Figure 1c). The BO outcomes succeeded in improving yields of **1** in a small number of trials, as well reducing the amount of reagents usage, compared to conventional trial-and-error optimization (Figure 1b,c).

## 2. Results and Discussion

### 2.1. BO-Assisted Multiparameter Screening of Sulfurization of **2** in 2 g Scale and Scale-Up

The initial training set (ITS), four kinds of numeric synthetic parameters, and the corresponding yields NaSH aq. [(4.5–6.5 eq.), and the concentration of NaSH in water (7.4–14.8 M)], sulfur (1.2–3.0 eq.), the concentration of **2** in toluene (0.76–2.78 M) and the % GC yields of **1** (39–76%) in entries 2–7, Table 1 for BO were prepared with reference to second-generation synthetic conditions: concentration of **2** in toluene (1.71 M), the amount of aq. NaSH [6 eq., concentration in H_2_O (11.9 M)], sulfur (3 eq.), and 8.5 mol% of tetrabutylammonium bromide (TBAB) at 95 °C (the oil bath temperature) for 24 h, 100 mL 3 neck flask with stirrer bar (~600 rpm) (entry 1, Table 1).

To perform BO-assisted multiparameter screening using the ITS that consists of six trials (entries 2–7), GPyOpt (BO algorithms: freely downloadable from open-source project libraries) was used as the BO framework in Python [32]. During screening, BO was conducted using a single expected improvement (EI) as an acquisition function. Eventually, we determined the more appropriate conditions obtained by BO, including seven datapoints (entries 2–8) with NaSH = 5.8 eq., and 8.1 M in water, sulfur = 1.4 eq., and the concentration of **2** in toluene = 1.22 M, producing a 78% conversion yield, together with 12% recovery of **2**, as well as reducing the amount of sulfur (entries 1 and 8). Moreover, BO-assisted screening did not improve a conversion yield of **1**. To make an even more improvement of the conditions, LHS, known to be a statistical method for generating a nearly random sample of parameters from a multidimensional distribution, was employed as a sampling method for the ITS [33]; it is also known to be an efficient sampling technique that can provide accurate results with far fewer data points than that of simple random sampling [34]. In fact, LHS often displays a higher performance than random sampling in BO-assisted multiparameter screening for the organic synthetic conditions [18,20]. To obtain the initial parameters to be examined with LHS, we used pyDOE2 as the experimental design package in Python [35]. The LHS algorithm suggested statistically random five reaction conditions as well. The evaluation of these conditions afforded the corresponding conversion yields (31–68% in entries 9–13). Using the BO with the ITS consisting of the five trails (entries 9–13), the next parameter to examine was predicted in entry 14, leading to **1** in 70% yield. After two trials of the same protocol, the following parameters were identified: NaSH = 7.0 eq., and 29.8 M in water, sulfur = 1.3 eq., and the concentration of **2** in toluene = 4.54 M, resulting in **1** in 89% conversion yield with almost no recovery of **2** (less than 1%, entry 16). Having succeeded in exploring suitable synthetic conditions using BO, we next focused our attention on the scale-up reaction with twenty grams of starting material **2**. For the scale-up synthesis of **1**, we used the manufacturing apparatus consisting of a larger three-neck flask (volume: 500 mL) with reflux condenser and overhead stirrer motor. Gratifyingly, the conversion yield was almost the same (87%, entry 17) despite a 10-fold scale-up. This favorable result inspired us to further examine the scale-up to a hundred-gram scale. We found that our established conditions were applicable to the reaction using a hundred grams of **2** as the minimum manufacturing scale, leading to 81% conversion yield of **1** with full consumption of **2** (entry 18).

### 2.2. Proposed Reaction Mechanism

**Figure 2:**
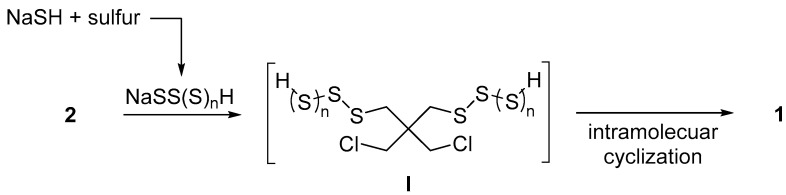
Assumed reaction mechanism.

Based on the previous relative work reported by Günther and Mautner [36], we proposed a reaction mechanism for the sulfurization/cyclization of **2** as shown in Figure 2. Initially, sodium sulfide reacts with sulfur to generate sodium oligomeric sulfide (n ≥ 0). Then, S_N_2-type sulfurization of **2** occurs with sodium oligomeric sulfide, followed by intramolecular cyclization of a key intermediate **I**, affording desired product **1**. In comparison to our optimized conditions by using conventional trial-and-error screening (entry 1, Table 1), the lower loading of toluene and H_2_O and the higher loading of NaSH appeared to play a critical role in yield improvement (entry 16). Reactive species **I** was efficiently generated in BO, suggesting a higher concentration. In contrast, a lower concentration and loading of reagents improved the yield (entry 8). The intramolecular cyclization of intermediate **I** preceded the intermolecular mechanism under the BO-suggested lower concentration. Moreover, the reduction in the amount of sulfur can be suppressed by high concentrations of reactive oligomeric sulfides, resulting in the diminution of undesired side products.

To gain further the insight into the suitable reaction conditions, the correlation coefficient between the four parameters and the conversion yield was estimated, because BO-assisted multiparameter screening cannot always suggest a critical factor in comparison to traditional exhaustive screening with the fixation of each parameter. Using the obtained results (entries 9–16, Table 1), the calculation of the correlation coefficient between the conversion yield of **1** and each loading of chemicals afforded the corresponding values (NaSH = +0.5, H_2_O = −0.6, sulfur = −0.4, toluene = −0.4, See the Supplementary Material). This result indicates a high concentration of NaSH in H_2_O, which can smoothly generate reaction species **I**, might play a more critical role in achieving a good conversion yield.

## 3. Materials and Methods

### 3.1. General Experimental Details

The samples were analyzed using gas chromatography (GC; Shimadzu gas chromatography GC-2014 equipped with a Shimadzu auto injector AOC-201). ^1^H- and ^13^ C-NMR spectra were recorded using a JEOL JNM-ECS400 (^1^H-NMR 400 MHz, ^13^C-NMR 100 MHz). ^1^H-NMR spectra are reported as follows: chemical shift in ppm relative to the chemical shift of CHCl_3_ at 7.26 ppm, integration, and multiplicities (d = doublet). ^13^C-NMR spectra were reported in ppm relative to the central line of the triplet for CDCl_3_ at 77 ppm. Tetrachloropentaerythrithiol was synthetized in Asahi Chemicals Co., Ltd. (Osaka, Japan) from pentaerthrithiol by Perstorp Chemical, Malmӧ, Sweden. TBAB was purchased by Lion specialty chemical. Toluene was obtained from Ando Chemicals. NaSH was purchased by Sankyou Kasei. Sulfur was purchased from Hosoi Kagaku Kougyou (Osaka, Japan). The NaClO solution was purchased from Nacalai Tesque, Inc. (Kyoto, Japan).

### 3.2. Typical Synthetic Procedure for Spiro-Dithiolane **1** in Two Grams Scale (Table 1, Entry 16)

After addition of NaSH aq. (7.0 eq., 66.5 mmol, 3.73 g, 29.8 M in water) to the three-neck flask (volume: 100 mL) with a reflux condenser, the reaction was stirred at 300 rpm for 10 min at 30 °C. Then, sulfur powder (1.3 eq., 12.4 mmol, 398 mg) was added to the three-neck flask (the generated H_2_S gas was externally trapped using a mixture of NaOH aq. (32 N, 100 mL)/NaClO aq. (10%, 100 mL)/water (300 mL)). The resulting reaction mixture was heated at 95 °C (the oil bath temperature) (90 °C: the reaction internal temperature). After stirring for 1 h at 90 °C (the reaction internal temperature), the reaction temperature was lowered to 50 °C. Tetrachloropentaerthriol (**2**, 9.5 mmol, 2 g) and tetrabutylammonium bromide (TBAB, 8.5 mol%, 0.26 g, 0.81 mmol) in toluene (6.8 mL) were added to the reaction mixture. After stirring for 24 h at 95 °C (the reaction internal temperature), the reaction mixture was cooled to room temperature. The crude organic phase (1.0 g) was diluted in toluene (2.3 mL). After washing of the obtained organic layer with water (1.0 mL), gas chromatography analysis (crude sample: 1.0 μL) was conducted, with 89% GC yields [37] as a yellow solid.

^1^H-NMR (400 MHz, CDCl_3_) *δ* 3.19 (d, *J* = 2.3 Hz, 8H) [29].

^13^C-NMR (100 MHz, CDCl_3_) *δ* 66.80, 47.78 [29].

### 3.3. Synthetic Procedure for Spiro-Dithiolane **1** in Twenty Grams Scale (Table 1, Entry 17)

After the addition of NaSH aq. (7.0 eq., 665 mmol, 37.3 g, 29.8 M in water) to the four-neck flask (volume: 1 L) with reflux condenser, the reaction was stirred at 210 rpm for 10 min at 40 °C. Then, sulfur powder (1.3 eq., 124 mmol, 3.98 g) was added to the four-neck flask (the generated H_2_S gas was externally trapped using a mixture of NaOH aq. (32 N, 200 mL)/NaClO aq. (10%, 200 mL)/water (300 mL)). The resulting reaction mixture was heated to 95 °C (the oil bath temperature) (90 °C: the reaction internal temperature). After stirring for 1 h at 90 °C (the reaction internal temperature), the reaction temperature was decreased to 50 °C. Tetrachloropentaerthriol (**2**, 95.0 mmol, 20 g) and tetrabutylammonium bromide (TBAB, 8.5 mol%, 2.6 g, 8.1 mmol) in toluene (68 mL) were added to the reaction mixture. After stirring for 24 h at 95 °C (the reaction internal temperature), the reaction mixture was cooled to room temperature. The crude organic phase (1.0 g) was diluted in toluene (2.3 mL). After washing of the obtained organic layer with water (1.0 mL), gas chromatography analysis (crude sample: 1.0 μL) was conducted, with 81% GC yields as a yellow solid [29].

### 3.4. Synthetic Procedure for Spiro-Dithiolane **1** in a Hundred Grams Scale (Table 1, Entry 18)

After addition of NaSH aq. (7.0 eq., 3.33 mol, 186.5 g, 29.8 M in water) to the four-neck flask (volume: 1 L) with reflux condenser, the reaction was stirred at 210 rpm for 30 min at 40 °C. Then, sulfur powder (1.3 eq., 0.62 mol, 19.9 g) was added to the four-neck flask (the generated H_2_S gas was externally trapped using a mixture of NaOH aq. (32 N, 400 mL)/NaClO aq. (10%, 400 mL)/water (600 mL)). The resulting reaction mixture was heated to 95 °C (the oil bath temperature) (90 °C: the reaction internal temperature). After stirring for 1 h at 90 °C (the reaction internal temperature), the reaction temperature was reduced to 50 °C. Tetrachloropentaerthriol (**2**, 0.475 mol, 100 g) and tetrabutylammonium bromide (TBAB, 8.5 mol%, 13.0 g, 0.04 mol) in toluene (340 mL) were added to the reaction mixture. After stirring for 24 h at 95 °C (the reaction internal temperature), the reaction mixture was cooled to room temperature. The crude organic phase (1.0 g) was diluted in toluene (2.3 mL). After washing of the obtained organic layer with water (1.0 mL), gas chromatography analysis (crude sample: 1.0 μL) was conducted, with 81% GC yields as a yellow solid [29].

### 3.5. Code: Latin Hypercube Sampling (LHS) Using pyDOE2

import pyDOE2 as doefrom sklearn.preprocessing import MinMaxScalerimport pandas as pdimport random

boundary = pd.DataFrame({‘paramA’:[2,9],’paramB’:[0.4,3.2],’paramC’:[0.6,3.6],’paramD’:[0.5,6.5]})

lhs = doe.lhs(4, samples = 5, criterion = ‘center’, random_state = 1)

scaler = MinMaxScaler()min_max = scaler.fit(boundary)

value = pd.DataFrame(min_max.inverse_transform(lhs), columns=boundary.columns)

print(value)

### 3.6. Code: Bayesian Optimization Using GPyOpt for Entries 2–8 in Table 1

import numpy as npimport GPyimport GPyOpt


X = np.array([[6.5, 1.2, 2.4, 3.5],

[4.5, 2.4, 2.4, 1.5],

[5.5, 1.2, 3.0, 5.5],

[5.5, 2.4, 1.2, 3.5],

[6.5, 1.8, 3.0, 1.5],

[4.5, 1.8, 1.2, 5.5],

[5.8, 2.2, 1.9, 3.4],

[6.2, 2.5, 1.5, 3.2]])
Y = -np.array([65, 51, 39, 76, 66, 52, 78, 74])[:, np.newaxis]





initial_x = Xinitial_y = Y





bounds = [{‘name’: ‘current’, ‘type’: ‘continuous’, ‘domain’: (2,9)},
{‘name’: ‘Init_molarity’, ‘type’: ‘continuous’, ‘domain’: (0.4,3.2)},
{‘name’: ‘electrolyte’, ‘type’: ‘continuous’, ‘domain’: (0.6,3.6)},
{‘name’: ‘time’, ‘type’: ‘continuous’, ‘domain’: (0.5,6.5)}]

















myBopt = GPyOpt.methods.BayesianOptimization(f=None,

domain=bounds,

X = initial_x,

Y = initial_y,

acquisition_type=‘EI’,













)





next_x = myBopt.suggest_next_locations()print(next_x)

### 3.7. Code: Bayesian Optimization Using GPyOpt for Entries 9–16 in Table 1

import numpy as npimport GPyimport GPyOpt

X = np.array([[5.5, 2.36, 2.1, 1.1],

[2.7, 1.8, 3.3, 2.3],

[6.9, 0.68, 2.7, 3.5],

[8.3, 2.92, 0.9, 5.9],

[4.1, 1.24, 1.5, 4.7],

[6.7, 0.77, 2.7, 3.3],

[6.5, 1.36, 2.0, 2.2]])
Y = -np.array([67, 31, 68, 48, 66, 70, 78])[:, np.newaxis]



initial_x = X

initial_y = Y





bounds = [{‘name’: ‘current’, ‘type’: ‘continuous’, ‘domain’: (2,9)},
{‘name’: ‘Init_molarity’, ‘type’: ‘continuous’, ‘domain’: (0.4,3.2)},
{‘name’: ‘electrolyte’, ‘type’: ‘continuous’, ‘domain’: (0.6,3.6)},
{‘name’: ‘time’, ‘type’: ‘continuous’, ‘domain’: (0.5,6.5)}]











myBopt = GPyOpt.methods.BayesianOptimization(f=None,

domain=bounds,

X = initial_x,

Y = initial_y,

acquisition_type=‘EI’,





)

next_x = myBopt.suggest_next_locations()print(next_x)

### 3.8. Code: Calculation of Correlation Coefficient Using Pandas

import pandas as pdimport seaborn as snsimport matplotlib.pyplot as pltfrom matplotlib import rcParams


NaSH = [5.5, 2.7, 6.9, 8.3, 4.1, 6.7, 6.5, 7.0] H2O = [2.36, 1.8, 0.68, 2.92, 1.24, 0.77, 1.36, 0.42] sulfur = [2.1, 3.3, 2.7, 0.9, 1.5, 2.7, 2.0, 1.3]toluene = [1.1, 2.3, 3.5, 5.9, 4.7, 3.3, 2.2, 1.1] result = [67, 31, 68, 48, 66, 70, 78, 89]


df = pd.DataFrame({“NaSH”:NaSH,
            “H2O”:H2O,
            “sulfur”:sulfur,
            “toluene”:toluene,
            “result”:result,
            })


sns.heatmap(df.corr(), cmap=“magma”, annot=True, fmt=“1.1f”)

## 4. Conclusions

In conclusion, ML-assisted screening of the reaction conditions was conducted for the synthesis of spiro-dithiolane **1**, which is a key intermediate of polyvalent mercaptans. The suitable reaction conditions on a 2-gram scale were rapidly estimated through BO with a small number of experiments within seven trials including ITS, resulting in a 78% conversion yield. Moreover, the sampling of ITS using LHS resulted in finding the better conditions (89% conversion yield) in comparison with the conventional trial-and-error screening and random screening. The established conditions were applicable to a hundred grams scale synthesis, affording desired product **1** in 81% GC yield. Comparing the newly and previously established conditions of the present sulfurization process, we found that the concentration of the reagents played a key role in achieving a suitable yield of **1**. Further application of BO in other large-scale syntheses is ongoing in our group.

## Figures and Tables

**Table 1:** BO-assisted multiparameter screening of the sulfurization of pentaerythrityl tetrachloride **2** to spiro-dithiolane **1** in two grams scale (entries 2–8: BO based on ITS by random sampling; entries 9–16: BO based on ITS by LHS and scale-up (entry 17: twenty grams scale, entry 18: a hundred grams scale).

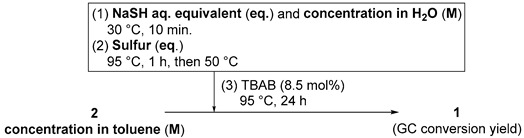						
Entry	NaSH aq.		Sulfur (eq.)	2 Concentration in Toluene (M)	%Conversion Yield (% GC Yield of 1)	Note
	(**eq.**)	**Concentration in H_2_O** (**M**)				
1	6.0	11.9	3.0	1.71	68	Previous optimized conditions
2	6.5	14.8	2.4	1.19	65	ITS prepared by random sampling
3	4.5	7.4	2.4	2.78	51	
4	5.5	14.8	3.0	0.76	39	
5	5.5	7.4	1.2	1.19	76	
6	6.5	9.8	2.0	2.78	66	
7	4.5	9.8	1.2	0.76	52	
8	5.8	8.1	1.4	1.22	78 ^a^	Estimated conditions by BO and entries 2–7
9	5.5	5.3	2.1	4.54	67	ITS prepared by LHS
10	2.7	7.0	3.3	2.16	31	
11	6.9	18.4	2.7	1.42	68	
12	8.3	4.3	0.9	0.84	48	
13	4.1	10.0	1.5	1.06	66	
14	6.7	16.2	2.7	1.50	70	Estimated conditions by BO and entries 9–13
15	6.5	9.2	2.0	2.26	78	Estimated conditions by BO and entries 9–14
16	7.0	29.8	1.3	4.54	89 ^b^	Estimated conditions by BO and entries 9–15
17	7.0	29.8	1.3	4.54	87 ^b^	20 g of **2** Overhead stirrer motor (210 rpm) based on entry 16
18	7.0	29.8	1.3	4.54	81 ^b^	100 g of **2** Overhead stirrer motor (210 rpm) based on entry 16

^a^ 12% of **2** was recovered. ^b^ **2** was fully consumed.

## Data Availability

The data presented in this study are available upon request.

## Supplementary Materials

The following supporting information can be downloaded at: https://www.mdpi.com/article/10.3390/molecules28135180/s1, Table S1: BO-assisted multiparameter screening of sulfurization; Calculation of GC yield from GC conversion chart: Table 1, entry 18 (100 grams scale); Figure of calculation of the correlation coefficient between the conversion yield of 1 and each loading of chemicals afforded the corresponding values.

## Sample Availability

Samples of compound **1** are available from the corresponding author upon reasonable request.
