# Supplementary material for: Bayesian Optimization-Assisted Screening to Identify Improved Reaction Conditions for Spiro-Dithiolane Synthesis
Source: Molecules. 2023 Jul 3;28(13):5180. doi: 10.3390/molecules28135180 (PMC10343712; doi:10.3390/molecules28135180)

# Bayesian Optimization-assisted Screening to Identify Improved Reaction Conditions for Spiro-dithiolane Synthesis

Masaru Kondo,[1,2] H. D. P. Wathsala,[1] Kazonori Ishikawa,[1] Daisuke Yamashita,[3] , Takeshi Miyazaki,[3] Yoji Ohno,[3] Hiroaki Sasai,[1] Takashi Washio,[1] and Shinobu Takizawa*[1]

[1]*SANKEN, Osaka University, Mihogaoka, Ibaraki-shi, Osaka 567-0047, Japan*

[2]*Department of Materials Science and Engineering, Graduate School of Science and Engineering, Ibaraki University, Nakanarusawa-cho, Hitachi-shi, Ibaraki 316-8511, Japan*

[3]*Asahi Chemical Co. Ltd., Yodogawa-ward, Osaka 532-0035, Japan*

[4]*Graduate School of Pharmaceutical Sciences, Osaka University, Suita-shi, Osaka 565-0871, Japan*

E-mail: taki@sanken.osaka-u.ac.jp

## Table of Contents

1 Optimization table S 1

2 GC yield calculation S 3

3 Calculation of correlation coefficient using pandas S 6

## 1. BO-assisted multiparameter screening of sulfurization

As a result, we can get many advantages from them in order to enhance Asahi`s products demand.

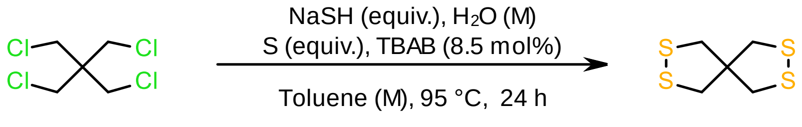


**1** (2 gram) **2**

Optimization Table S1: As a result, we can get many advantages from them in order to enhance Asahi`s products demand.

| entry | NaSH (eq.) | $H_2O$ (M) | S (eq.) | Toluene (M) | GC conv.% |
|---|---|---|---|---|---|
| 1 | 6.0 | 1.38 | 3.0 | 2.36 | 68 |
| 2 | 6.5 | 1.67 | 2.4 | 1.42 | 65 |
| 3 | 4.5 | 1.21 | 2.4 | 3.32 | 51 |
| 4 | 5.5 | 1.98 | 3.0 | 0.9 | 39 |

| | | | | | |
|---|---|---|---|---|---|
| 5 | 5.5 | 0.99 | 1.2 | 1.42 | 76 |
| 6 | 6.5 | 1.11 | 2.0 | 3.32 | 66 |
| 7 | 4.5 | 1.61 | 1.2 | 0.9 | 52 |
| 8 | 5.8 | 1.02 | 1.4 | 1.46 | 78 |
| 9 | 5.5 | 5.3 | 2.1 | 4.54 | 67 |
| 10 | 2.7 | 7.0 | 3.3 | 2.16 | 31 |
| 11 | 6.9 | 18.4 | 2.7 | 1.42 | 68 |
| 12 | 8.3 | 4.3 | 0.9 | 0.84 | 48 |
| 13 | 4.1 | 10.0 | 1.5 | 1.06 | 66 |
| 14 | 6.7 | 16.2 | 2.7 | 1.5 | 70 |
| 15 | 6.5 | 9.2 | 2.0 | 2.26 | 78 |
| 16 | 7.0 | 29.8 | 1.3 | 4.54 | 89 |
| 17 | 7.0 | 29.8 | 1.3 | 4.54 | 87[a] |
| 19 | 7.0 | 29.8 | 1.3 | 4.54 | 81[b] |

Reaction condition: **1**(2gram), NaSH (eq), Sulphur (eq), $H_2O$ (M), Toluene (M), TBAB (8.5 mol %) at 95 °C for 24 h; a) 20g scale; b) 100g scale

*Calculation of Molarity (M)*:

$H_2O$ (M): mmol of **1**/$H_2O$ volume

Toluene (M): mmol of **1**/Toluene volume

$H_2O$ Volume: $H_2O$ weight/density

Toluene volume: Toluene weight/density

$H_2O$ weight: WR1*NaSH weight

Toluene weight: WR2* weight of **1**

## 2. Calculation of GC yield from GC conversion chart: Table 2, entry 03 (100g scale)

a) Pure GC chart of tetrachloropentaerythritol (4Cl): **8:4 min** (1.46 mol/L)

Chromatogram

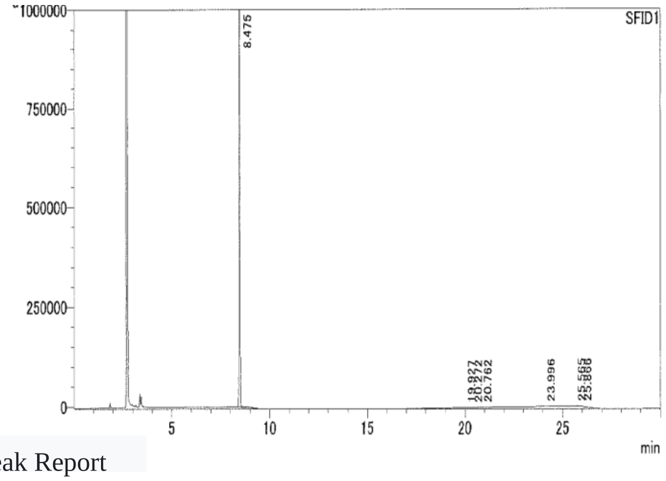


Peak Report

| Peak# | tR (min) | Area | Hight | Area% | Mark | Compound name |
|---|---|---|---|---|---|---|
| 1 | 8.475 | 4693741 | 1609896 | 99.691 | | |
| 2 | 19.927 | 1321 | 110 | 0.028 | | |
| 3 | 20.272 | 1660 | 112 | 0.035 | V | |
| 4 | 20.762 | 2303 | 175 | 0.049 | V | |
| 5 | 23.996 | 1689 | 311 | 0.036 | | |
| 6 | 25.565 | 1678 | 280 | 0.036 | V | |
| 7 | 25.866 | 5912 | 394 | 0.126 | | |
| 合計 | | 4708304 | 1611278 | 100.000 | | |

b) Pure GC chart of Spiro-dithiolane (4S): **15.8 min** (1.46 mol/L)

Chromatogram

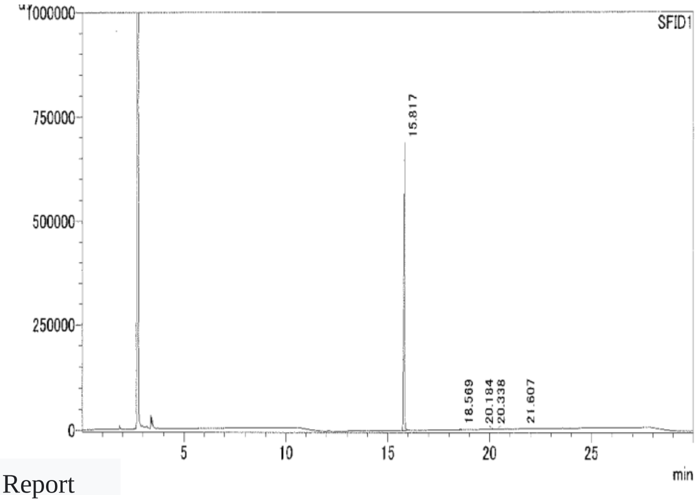


Peak Report

| Peak# | tR (min) | Area | Hight | Area% | Mark | Compound name |
|---|---|---|---|---|---|---|
| 1 | 15.817 | 2328234 | 682949 | 99.391 | | |
| 2 | 18.569 | 9123 | 1594 | 0.389 | | |
| 3 | 20.184 | 2535 | 147 | 0.108 | V | |
| 4 | 20.338 | 1571 | 187 | 0.067 | V | |
| 5 | 21.607 | 1042 | 109 | 0.044 | | |
| 合計 | | 2342505 | 684987 | 100.000 | | |

b) 1:1 ratio of tetrachloropentaerythritol (50%) and Spiro-dithiolane (50%): (1.46 mol/L)

Chromatogram

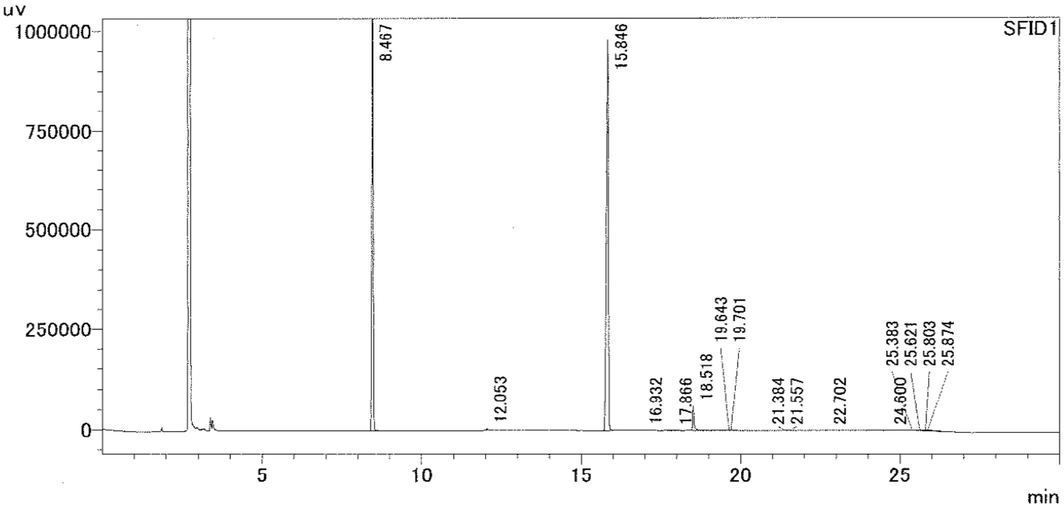


Peak Report

| Peak# | tR (min) | Area | Hight | Area% | Mark | Compound name |
|---|---|---|---|---|---|---|
| 1 | 8.467 | 3296188 | 1237215 | 42.466 | | |
| 2 | 12.053 | 21424 | 4619 | 0.276 | | |
| 3 | 15.846 | 4107187 | 974362 | 52.915 | S | |
| 4 | 16.932 | 14960 | 938 | 0.193 | V | |
| 5 | 17.866 | 4785 | 286 | 0.062 | V | |
| 6 | 18.518 | 214134 | 60164 | 2.759 | SV | |
| 7 | 19.643 | 1352 | 336 | 0.017 | TV | |
| 8 | 19.701 | 4285 | 389 | 0.055 | TV | |
| 9 | 21.384 | 4557 | 808 | 0.059 | | |
| 10 | 21.557 | 1240 | 221 | 0.016 | V | |
| 11 | 22.702 | 1269 | 244 | 0.016 | V | |
| 12 | 24.600 | 1173 | 107 | 0.015 | | |
| 13 | 25.383 | 3226 | 435 | 0.042 | V | |
| 14 | 25.621 | 21606 | 1452 | 0.278 | V | |
| 15 | 25.803 | 10260 | 1884 | 0.132 | V | |
| 16 | 25.874 | 54273 | 2139 | 0.699 | V | |
| 合計 | | 7761918 | 2285598 | 100.000 | | |

d) Draw a graph GC area vs GC yield) Graph of GC area vs GC yield

X = GC area (-)

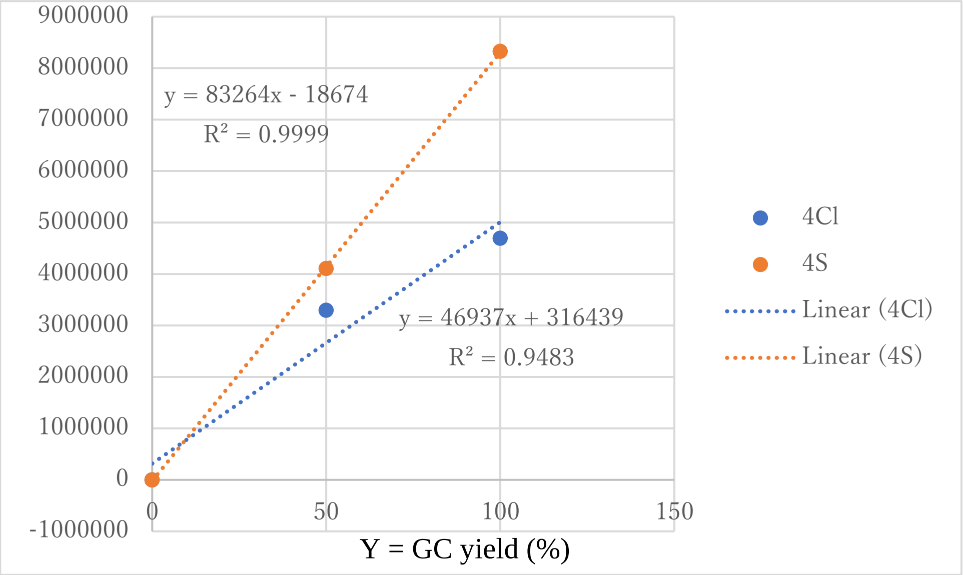

e) 100g scale GC chart (Table 2, entry 3): (1.46 mol/L)

Chromatogram

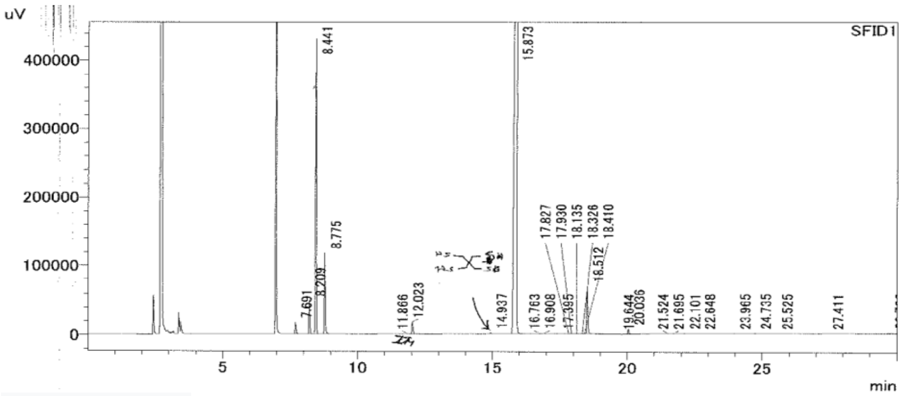


Peak Report

| Peak# | tR (min) | Area | Hight | Area% | Mark | Compound name |
|---|---|---|---|---|---|---|
| 1 | 7.691 | 48635 | 16815 | 0.567 | M | |
| 2 | 8.209 | 118017 | 47676 | 1.376 | | |
| 3 | 8.441 | 992121 | 426902 | 11.570 | V | |
| 4 | 8.775 | 279507 | 116476 | 3.260 | | |
| 5 | 11.866 | 3422 | 674 | 0.040 | | |
| 6 | 12.023 | 57992 | 17445 | 0.676 | V | |
| 7 | 14.937 | 11490 | 1822 | 0.134 | | |
| 8 | 15.873 | 6758659 | 1310948 | 78.817 | S | |
| 9 | 16.763 | 1734 | 276 | 0.020 | TV | |
| 10 | 16.908 | 6155 | 619 | 0.072 | TV | |
| 11 | 17.395 | 1080 | 278 | 0.013 | | |
| 12 | 17.827 | 1623 | 233 | 0.019 | V | |
| 13 | 17.930 | 3189 | 325 | 0.037 | V | |
| 14 | 18.135 | 5747 | 470 | 0.067 | V | |
| 15 | 18.326 | 4410 | 649 | 0.051 | V | |
| 16 | 18.410 | 2894 | 747 | 0.034 | V | |
| 17 | 18.512 | 208957 | 68043 | 2.437 | V | |
| 18 | 19.644 | 2883 | 818 | 0.034 | | |
| 19 | 20.036 | 25712 | 7748 | 0.300 | | |
| 20 | 21.524 | 1416 | 396 | 0.017 | V | |
| 21 | 21.695 | 1199 | 289 | 0.014 | | |
| 22 | 22.101 | 4088 | 951 | 0.048 | | |

Area of **6758659** = GC yield??

Y= GC area (-)

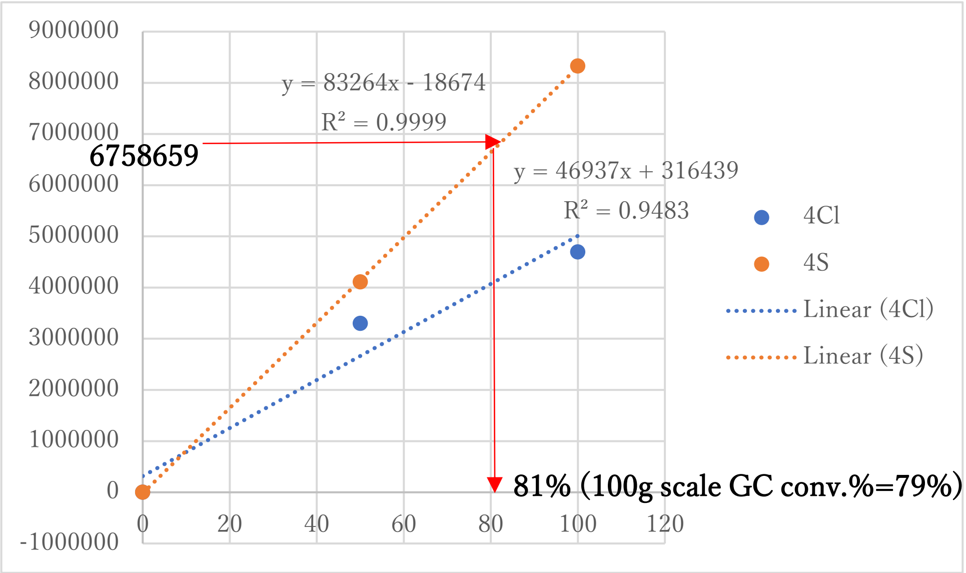

*Calculation [1]:*

$$Y = 83264X\text{-}18674$$

$$6758659+18674/83264 = X$$

$$X = 81\%$$

**GC conversion yield ≈ GC calculated yield±2**

Reference

1. https://www.shimadzu.com/an/service-support/technical-support/analysis-basics/fundamentals/results.html

**3. Calculation of the correlation coefficient between the conversion yield of 1 and each loading of chemicals afforded the corresponding values.**

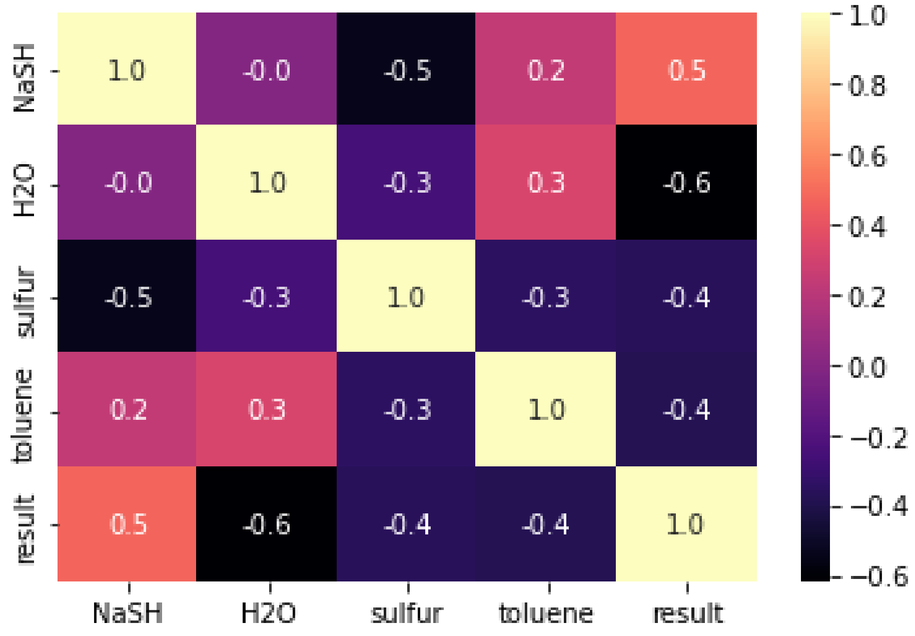

Supplement: Supplementary file 1 [file molecules-28-05180-s001.zip › molecules-2451165-supplementary.pdf]
